# Effect of Adding Apolipoprotein B Testing on the Prevalence of Dyslipidemia and Risk of Cardiovascular Disease in the Korean Adult Population

**DOI:** 10.3390/metabo14030169

**Published:** 2024-03-18

**Authors:** Rihwa Choi, Sang Gon Lee, Eun Hee Lee

**Affiliations:** 1 Department of Laboratory Medicine, Green Cross Laboratories, Yongin 16924, Republic of Korea; pirate0720@naver.com; 2 Department of Laboratory Medicine and Genetics, Samsung Medical Center, Sungkyunkwan University School of Medicine, Seoul 06351, Republic of Korea; 3 Green Cross Laboratories, Yongin 16924, Republic of Korea

**Keywords:** apolipoprotein B, ApoB, cutoff, dyslipidemia, lipid, Republic of Korea

## Abstract

Traditional lipid parameters—including total cholesterol (TC), triglycerides (TG), low-density lipoprotein cholesterol (LDL-C), high-density lipoprotein cholesterol (HDL-C), and non-HDL-C (calculated as TC minus HDL-C)—have long been used as indicators of cardiovascular disease (CVD) risk. The laboratory records of 9604 Korean adults who underwent traditional lipid panel tests (TC, TG, and HDL), as well as ApoB testing, were analyzed to evaluate the prevalence of dyslipidemia and high CVD risk (utilizing the NCEP ATP III criteria for traditional lipid panels and various ApoB test cutoffs recommended by international guidelines (145 mg/dL, 130 mg/dL, and 100 mg/dL)). The overall prevalence of dyslipidemia, as determined by traditional lipid panel criteria, was 27.4%. Utilizing the ApoB cutoffs of 145 mg/dL, 130 mg/dL, and 100 mg/dL resulted in prevalence figures of 5.3%, 11.0%, and 36.3%, respectively. The concordance in dyslipidemia classification between traditional lipid tests and ApoB at cutoffs of 145 mg/dL, 130 mg/dL, and 100 mg/dL was 78.4%, 81.3%, and 74.7%, respectively. Up to 17.5% of participants, based on an ApoB cutoff of ≥100 mg/dL, exhibited isolated high ApoB in the absence of traditional lipid test anomalies. Incorporating ApoB testing could enhance the identification of Koreans at high CVD risk.

## 1. Introduction

Cardiovascular disease (CVD) remains a leading cause of morbidity and mortality worldwide, presenting a significant public health challenge [1,2]. The traditional approach to assessing the risk of CVD has relied heavily on evaluating conventional lipid profiles, including total cholesterol (TC), low-density lipoprotein cholesterol (LDL-C), high-density lipoprotein cholesterol (HDL-C), and triglycerides (TG) [2,3]. However, this method does not capture the complete spectrum of lipid-related CVD risk [3,4]. Recent advancements in lipidology have highlighted the importance of Apolipoprotein B (ApoB) as a superior marker for atherogenic lipoprotein particles [3,5,6,7,8,9,10]. ApoB is a principal component of all potentially atherogenic lipoproteins, including very-low-density lipoprotein (VLDL), intermediate-density lipoprotein (IDL), LDL, and lipoprotein(a) [5,6,9]. Each of these particles contains one ApoB molecule, making its plasma concentration a direct measure of the total atherogenic particle number [9,10]. Consequently, ApoB has emerged as a significant predictor of CVD risk, potentially offering a more accurate risk stratification than traditional lipid measurements alone [3,8,11].

In the Korean population, where the prevalence of dyslipidemia and its consequent impact on CVD risk is a growing concern, there is a pressing need to explore more effective screening strategies [12,13,14]. The incorporation of ApoB testing into routine lipid panels could represent a pivotal shift in identifying individuals at elevated risk for CVD [3,13,14]. However, the standardization of the ApoB test, which aims to ensure that ApoB test results are comparable across different laboratories and analytical platforms to enhance the reliability of ApoB as a biomarker for CVD risk assessment and management, is still ongoing [3,15,16,17]. The lack of standardized analytical methods for ApoB tests has necessitated the assessment of different reference intervals across various analytical methods and populations [6,15,16]. Differences in cutoffs and reference intervals from various international guidelines for ApoB have been suggested, yet, there are no uniform cutoffs for ApoB in the Korean population [3,18,19]. Moreover, the impact of adding ApoB testing to the assessment of dyslipidemia prevalence and CVD risk in the Korean population has been less explored compared to Western populations [6,13].

Therefore, this study aimed to investigate the impact of incorporating ApoB testing into traditional lipid assessments on the prevalence of dyslipidemia and high CVD risk in the Korean adult population, as well as the changes in high CVD risk prevalence as categorized by lipid tests using various ApoB cutoffs recommended by international clinical guidelines. By examining the implications of this approach, the study seeks to provide valuable insights into optimizing CVD risk assessment and prevention strategies for this demographic.

## 2. Materials and Methods

### 2.1. Study Population

This study was conducted as a retrospective, observational analysis of data obtained from Korean adults who visited local clinics and hospitals and underwent both traditional lipid panel tests (TC, TG, and HDL-C) and ApoB testing from 1 January to 31 December 2021. Data were collected from the laboratory information system records of Green Cross Laboratories, one of Korea’s largest referral laboratories, which provides lipid testing services to local clinics and hospitals. The inclusion criteria were adults aged 20 years and older who had been tested for all four lipid parameters. The exclusion criteria included individuals with incomplete information on age, sex, and lipid profiles. Since non-HDL-C and LDL-C were calculated using TC, TG, and HDL-C for this study, results with negative values for non-HDL-C and LDL-C were excluded. The study aimed to investigate the impact of adding ApoB testing to traditional lipid tests, focusing on the prevalence of subjects identified as high-risk for CVD based on lipid test results; therefore, duplicate test results for the same individuals were excluded.

### 2.2. Laboratory Measurements

All laboratory tests were conducted at Green Cross Laboratories. Traditional lipid tests, including TC, TG, HDL-C, and ApoB, were carried out using the Cholesterol Gen.2 (Roche, Mannheim, Germany), Triglyceride reagent without glycerol blank (Roche, Mannheim, Germany), HDL-Cholesterol plus 3rd generation kits, and the Tina-quant Apolipoprotein B ver.2 reagent kit (Roche, Mannheim, Germany), which is traceable to the IFCC reference material SP3-07. These tests were performed using automated Cobas 8000 system c702 analyzers (Roche, Mannheim, Germany) [18,20]. Non-HDL-C was calculated using the formula TC minus HDL-C. LDL-C was calculated using three equations suggested by Friedewald et al. [21], Martin et al. [22], and Sampson et al. [23], referred to in this study as the Friedewald equation, the Martin/Hopkins equation, and the Sampson/NIH equation, respectively. The accuracy of the traditional lipid profile tests (TC, TG, HDL-C) was verified through participation in accuracy-based external quality assurance programs such as the Lipids Standardization Program by the Centers for Disease Control, USA; the ABL surveys by the College of American Pathologists; and the Korean External Quality Assessment Scheme [24] during the study period. Although there was no accuracy-based external quality assurance program specifically for the ApoB test due to the lack of assay standardization, participation in external quality programs as mentioned above was also undertaken.

### 2.3. Definition

Dyslipidemia was defined according to the criteria of the National Cholesterol Education Program Adult Treatment Panel III (NCEP ATP III) [25]. Results with TC ≥ 240 mg/dL, TG ≥ 200 mg/dL, non-HDL-C ≥ 190 mg/dL, HDL-C < 40 mg/dL, or calculated LDL-C ≥ 160 mg/dL (based on any of the three equations) were classified as dyslipidemia. High ApoB, indicative of a high risk of CVD, was categorized using three cutoffs recommended by the following international guidelines (Canadian, American, and European guidelines, as well as specific treatment target guidelines): ApoB ≥ 145 mg/dL as suggested by the Canadian Heart Association guidelines to define a high risk of CVD [26], ApoB ≥ 130 mg/dL as recommended by the American College of Cardiology and American Heart Association (ACC/AHA, USA) to define a high risk of CVD [5,27], and ApoB ≥ 100 mg/dL as suggested by various societies, including the European Society of Cardiology and European Atherosclerosis Society (ESC/EAS), as a treatment target level for dyslipidemia [11,27,28].

### 2.4. Statistical Analysis

Descriptive statistics were employed to summarize the demographic and clinical characteristics of the study population. For age and lipid test results, which were not normally distributed, non-parametric Mann–Whitney tests were used for comparisons by sex. The prevalence of dyslipidemia was determined based on traditional lipid panels and ApoB levels, in accordance with the NCEP ATP III criteria and international guidelines, respectively. The agreement in dyslipidemia classification between traditional lipid tests and ApoB at various cutoffs was evaluated using 2 × 2 contingency tables. Statistical analyses were conducted using MedCalc Statistical Software Version 20.116 (MedCalc Software Ltd., Ostend, Belgium; https://www.medcalc.org; accessed on 7 February 2024). *p*-values of less than 0.05 were considered statistically significant.

## 3. Results

During the one-year study period, 9604 Korean adults (4427 men and 5177 women) with a median age of 58.7 years (interquartile range, IQR 47.5–66.8) were included in the analysis. The lipid test results are summarized in Table 1. Although all variables, including age, TC, TG, HDL-C, non-HDL-C, and calculated LDL-C, showed significant differences between men and women (all *p* < 0.05), there were no significant differences in ApoB levels between the sex groups (*p* = 0.7865).

**Figure 1:**
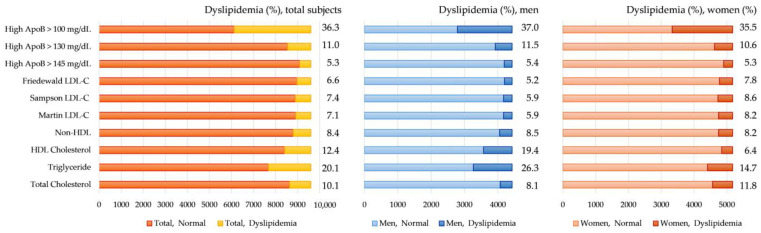
Prevalence of dyslipidemia. Dyslipidemia is defined by any of the following lipid test results: TC ≥ 240 mg/dL, TG ≥ 200 mg/dL, non-HDL-C ≥ 190 mg/dL, HDL-C < 40 mg/dL, or calculated LDL-C ≥ 160 mg/dL. Additionally, ApoB levels are evaluated using three cutoffs: ≥145 mg/dL, ≥130 mg/dL, and ≥100 mg/dL.

The prevalence of dyslipidemia based on lipid test results according to the NCEP ATP III criteria and high ApoB according to three cutoffs (≥145 mg/dL, ≥130 mg/dL, and ≥100 mg/dL) are summarized in Figure 1. The overall prevalence of dyslipidemia, defined by any of the conditions of high TC, high TG, low HDL-C, high non-HDL-C, or high calculated LDL-C, was 27.4% for all subjects. The prevalence of dyslipidemia based on traditional lipid test results was significantly different between men and women (all Ps < 0.0001). However, the prevalence of high ApoB did not significantly differ between men and women for any of the three cutoffs. The prevalence of hypercholesterolemia (high TC ≥ 240 mg/dL) and hyper-LDL-cholesterolemia (calculated LDL-C ≥ 160 mg/dL) was significantly higher in women than in men, whereas hypertriglyceridemia (TG ≥ 200 mg/dL), hyper-non-HDL-cholesterolemia (non-HDL-C ≥ 190 mg/dL), and hypo-HDL-cholesterolemia (HDL-C < 40 mg/dL) were significantly higher in men than in women (all Ps < 0.0001). The overall prevalence of high ApoB was 5.3% for ≥145 mg/dL, 11.0% for ≥130 mg/dL, and 36.3% for ≥100 mg/dL, respectively.

The agreement in dyslipidemia classification based on traditional lipid tests and ApoB results, using three cutoffs, was assessed with a 2 × 2 contingency table and summarized in Table 2. The highest overall percent agreement was observed at the ApoB ≥ 130 mg/dL cutoff, recommended by the ACC/AHA and multi-society guidelines, with an agreement rate of 80.5% (95% confidence interval [CI]: 79.7–81.3%), while the lowest overall percent agreement occurred at the ApoB ≥ 100 mg/dL cutoff, suggested by multi-society guidelines as a treatment target, with an agreement rate of 73.9% (95% CI: 73.0–74.7%). The highest positive percent agreement was found at the ApoB ≥ 145 mg/dL cutoff (96.3%, 95% CI: 94.3–97.6), as recommended by the Canadian guidelines for defining high CVD risk, and the highest negative percent agreement was noted at the ApoB ≥ 100 mg/dL cutoff (86.5%, 95% CI: 85.6–87.3%).

**Figure 2:**
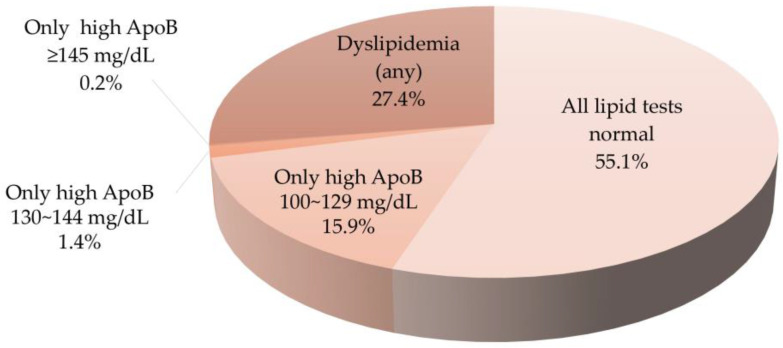
The additional value of apolipoprotein B (ApoB) in traditional lipid testing. Dyslipidemia is classified by the presence of any of the following lipid test results: TC ≥ 240 mg/dL, TG ≥ 200 mg/dL, non-HDL-C ≥ 190 mg/dL, HDL-C < 40 mg/dL, or calculated LDL-C ≥ 160 mg/dL—determined by any of the three applicable equations—with or without elevated ApoB. About 17.5% of all subjects were identified as high-risk for CVD solely based on elevated ApoB levels (≥100 mg/dL), as their other traditional lipid parameters were within normal ranges.

The additional value of the ApoB test alongside traditional lipid tests is demonstrated by assessing the proportion of patients not diagnosed with dyslipidemia through traditional lipid tests but identified as high-risk for CVD due to elevated ApoB levels, as presented in Figure 2. Of all the subjects, 5293 (55.1%) had normal lipid results for TC, TG, HDL-C, non-HDL-C, calculated LDL-C, and ApoB. A total of 2629 (27.4%) were classified as having dyslipidemia based on traditional lipid tests and/or ApoB results (any), while 1682 (17.5%) were identified as high-risk for CVD solely based on elevated ApoB levels (≥100 mg/dL) as their other traditional lipid parameters were within normal ranges. Among these 1682 subjects, 19 (0.2% of all subjects) had ApoB levels ≥ 145 mg/dL, 131 (1.4% of all subjects) had ApoB levels between 130 and 144 mg/dL, and 1532 (15.9%) had ApoB levels between 100 and 129 mg/dL. This indicates that the corresponding proportion of subjects identified as having a high risk of CVD based solely on ApoB levels of 39.0% using the ApoB cutoff of ≥100 mg/dL, 3.5% using the cutoff of ≥130 mg/dL, and 0.4% using the cutoff of ≥145 mg/dL among the 4311 subjects who did not have abnormal traditional lipid test results. The prevalence of isolated high ApoB was not significantly different between men and women at any of the three cutoffs (100, 130, 145 mg/dL).

## 4. Discussion

In this study, we assessed the impact of incorporating the ApoB test into traditional lipid tests through agreement analysis and by evaluating the prevalence of dyslipidemia and high ApoB using various cutoffs in Korean adults who underwent lipid testing at local clinics and hospitals. While the ApoB test has garnered attention in Western populations, its implications and the evaluation of ApoB cutoffs have been relatively less studied in the Korean population [6,18]. We determined the prevalence of dyslipidemia based on both traditional lipid tests and ApoB test results. The observed prevalence of dyslipidemia based on traditional lipid tests in this study was 27.4%, which is lower than the crude prevalence of 40.2% reported for the Korean general population between 2016 and 2020, according to the Korea National Health and Nutrition Examination Survey (KNHANES) data [12]. The prevalence of dyslipidemia identified using either traditional lipid tests or ApoB in this study (44.9%) was more aligned with the KNHANES findings [12]. The prevalence reported by KNHANES includes subjects treated with statins, whose lipid profile results fell below the cutoff due to the statin treatment [2,12,29,30]. Meanwhile, in the present study, clinical information on subjects, including statin treatment, was limited, which might result in lipid profile results below the cutoff. Given that current clinical guidelines in Korea recommend the ApoB test for specific groups like patients with diabetes mellitus and familial hypercholesterolemia, the observed differences in dyslipidemia prevalence may be influenced by the characteristics of the population studied [19,29,30]. The public database in Korea, provided by the Health Insurance Review and Assessment Service (HIRA), offers information on the usage of reimbursed diagnostic tests through specialized electronic data interchange (EDI) code. However, apolipoproteins (apolipoprotein A, B, C, E, and beta-lipoprotein) are collectively coded under one EDI code, D2630 [31]. According to the HIRA database, the annual number of patients tested for D2630 apolipoproteins has been increasing by about 10% annually, with 400,764 patients tested in 2021 [31]. This figure represents approximately 2.7% of the 14,990,233 patients who underwent D2611 total cholesterol tests in the same year, according to the database. Future studies are necessary to elucidate the clinical implications of ApoB testing to ensure its appropriate utilization in the Korean population.

In the present study, the prevalence of dyslipidemia based on traditional lipid tests was significantly different by sex, which aligns with previous findings in the Korean general population, as indicated by data from KNHANES and the Korean Dyslipidemia Fact Sheet [12]. However, ApoB levels and the prevalence of high ApoB did not significantly differ between sexes in this study. There have been inconsistent findings regarding ApoB levels by sex in the literature [18,32,33]. Serum lipid levels can be influenced by genetic background, hormonal changes, lifestyle factors, and healthcare behaviors, including access to healthcare facilities and adherence to physicians’ recommendations [2,32,33]. However, limited data exist on the ApoB test in the Korean population to determine which factors are associated with ApoB levels. Future studies are necessary to identify the factors linked to sex differences and similarities in lipids, including ApoB, within the Korean population. Future studies are needed to clarify the implications of sex on ApoB’s role in CVD risk prediction and management in the Korean population.

In this study, the overall percentage agreement between dyslipidemia identified through traditional lipid tests and the high risk of CVD determined by ApoB results, using various cutoffs, was highest with ApoB ≥ 130 mg/dL. The discordant percentage ranged from 20% to 60% of the total population in previous studies was comparable to the present study [34]. This cutoff is recommended by the ACC/AHA guidelines for the primary prevention of CVD to define high CVD risk. This level is close to the upper limit of reference intervals from previous studies in the Korean adult population and from the Framingham Offspring Study [18,27,35]. However, a recent study conducted in the Korean population, which utilized automated analytical methods for traditional lipid tests on platforms other than Roche (e.g., Siemens), and used stored specimens for the ApoB test from the Korean Genome and Epidemiology Study cohort, found that ApoB ≥ 2.06 umol/L, as measured on the Roche platform, showed significant differences in 8-year CVD events among the study population [13]. Using the conversion factor, 2.06 μmol/L is approximately equivalent to 106 mg/dL, aligning with the cutoffs set by multi-society guidelines as treatment targets [5,11,28,36]. Future studies focusing on analytical method standardization and detailed histories, with longitudinally obtained information on CVD risk and events, are necessary to determine the optimal cutoff values for ApoB in the evaluation and management of patients at risk for CVD.

In this study, 39.0%, 3.5%, and 0.4% of subjects who were not identified as having dyslipidemia based on traditional lipid test results were newly recognized as having a high-risk for CVD when using ApoB cutoffs of 100 mg/dL, 130 mg/dL, and 145 mg/dL, respectively. These individuals may be underdiagnosed or undertreated because traditional lipid profiles might not reveal the extent of their CVD risk, potentially leading to delayed intervention [5,34,37]. Previous studies conducted in Western populations have reported that the discordant group, identified by traditional lipid tests such as TC, TG, HDL, non-HDL, and LDL versus isolated high ApoB, suggests that ApoB is a stronger predictor of CVD than the other markers [5,34,37]. This discordance is thought to be due to the molecular metabolic composition of lipid particles, which can be cholesterol-enriched or depleted, with ApoB serving as a structural protein for all atherogenic particles, including VLDL, IDL, LDL, and lipoprotein(a) [5,34]. Furthermore, comparison studies in Western populations have reported greater benefits from lowering ApoB levels than from lowering LDL levels [5,34,37]. Medications such as statins, proprotein convertase subtilisin/kexin type 9 (PCSK9) inhibitors, and ezetimibe have been suggested to lower both LDL-C and ApoB levels [5,34]. However, the treatment for isolated high ApoB levels requires further research across diverse populations and in various clinical settings [5,34,37]. The early identification of high-risk individuals could lead to effective interventions that prevent heart attacks, strokes, or other CVD events, potentially offsetting the upfront costs of ApoB testing with savings from reduced emergency treatments, hospitalizations, and chronic disease management [5]. The fact that a significant percentage of patients are classified as high-risk for CVD through ApoB testing, despite having normal traditional lipid levels, underscores the importance of a more nuanced approach to CVD risk assessment [3,5]. Given that the prevalence of high-risk CVD is dependent on the cutoff used and the clinical implications of ApoB in the Korean population are still emerging, future research and policy decisions must carefully balance the financial costs against the potential medical benefits to enhance patient care and public health outcomes [3].

The limitation of this study was the lack of clinical information related to dyslipidemia and CVD, including comorbidities, medications, and factors associated with sex differences in lipid levels [2,3,5]. Due to limited clinical information, it is not possible to follow up on cardiovascular events. Further research should be conducted on the Korean population in diverse clinical contexts, accompanied by prospective follow-up plans. However, the strength of this study lies in its practical approach, utilizing common practice guidelines including NCEP ATP III, ACC/AHA, ESC/EAS, and the Canadian Heart Association to investigate the prevalence of dyslipidemia [5,11,26,27]. The findings of this study could potentially be generalizable to clinical laboratories that perform lipid testing with automated analytical assays from Roche on samples from Korean adults.

## 5. Conclusions

In conclusion, we explored the impact of incorporating the ApoB test into traditional lipid testing among Korean adults visiting local clinics and hospitals. Given the substantial agreement between dyslipidemia identified through traditional lipid tests and high CVD risk indicated by ApoB levels, and the potential to identify more patients at risk with ApoB testing, this study highlights the significant role ApoB testing can play in improving the detection of individuals at high-risk for CVD within the Korean adult population, particularly those with normal results from traditional lipid panels. Integrating ApoB testing into routine lipid assessments could enhance CVD risk stratification, providing a more detailed approach to identifying individuals who might benefit from early interventions, even when traditional lipid profiles appear normal. Since cutoffs are determined based on their clinical applicability across various situations, further research should be conducted on the Korean population in diverse clinical contexts, accompanied by prospective follow-up plans. Future research should delve into the clinical implications of different ApoB cutoffs, emphasizing the need for comprehensive clinical data on dyslipidemia and CVD, along with assay standardization.

## Figures and Tables

**Table 1:** Characteristics of study population and lipid test results.

Characteristics	Total (*n* = 9604)	Men (*n* = 4427)	Women (*n* = 5177)	*p*-Value
Age, year, median	58.7 (47.5 to 66.8)	57.2 (46.1 to 66.4)	59.6 (49.1 to 67.1)	<0.0001
Total cholesterol, mg/dL	177.0 (151.0 to 208.0)	171.0 (144.0 to 201.0)	182.0 (157.0 to 212.0)	<0.0001
Triglyceride, mg/dL	122.0 (85.0 to 181.0)	139.0 (95.0 to 204.0)	111.0 (79.0 to 162.0)	<0.0001
HDL-C, mg/dL	53.0 (44.0 to 64.0)	48.0 (41.0 to 57.0)	58.0 (49.0 to 69.0)	<0.0001
Non-HDL-C, mg/dL	121.0 (96.0 to 151.0)	120.0 (93.0 to 152.0)	120.0 (97.0 to 151.0)	0.0217
Cal.LDL-C, Friedewald equation, mg/dL [21]	91.0 (69.0 to 120.0)	88.0 (64.0 to 115.0)	95.0 (73.0 to 124.0)	<0.0001
Cal.LDL-C, Martin/Hopkins equation, mg/dL [22]	96.0 (74.0 to 124.0)	94.0 (71.0 to 121.0)	98.0 (77.0 to 126.0)	<0.0001
Cal.LDL-C, Sampson/NIH equation, mg/dL [23]	95.0 (73.0 to 126.0)	92.0 (68.0 to 119.0)	98.0 (76.0 to 127.0)	<0.0001
Apolipoprotein B, mg/dL	89.7 (73.6 to 110.7)	90.0 (72.9 to 111.4)	89.3 (74.0 to 110.0)	0.7865

Data are presented as median and interquartile range. Abbreviations: Cal. calculated.

**Table 2:** Agreement of dyslipidemia classification using a 2 × 2 contingency table.

ApoB Cutoff	Dyslipidemia in Traditional Lipid Test (Any)	Total Subjects (*n* = 9604)			Men (*n* = 4427)			Women (*n* = 5177)		
		High ApoB (*n*)	Normal (*n*)	Total (*n*)	High ApoB (*n*)	Normal (*n*)	Total (*n*)	High ApoB (*n*)	Normal (*n*)	Total (*n*)
ApoB ≥ 100 mg/dL	Dyslipidemia (*n*)	1800	829	2629	879	490	1369	921	339	1369
	Normal (*n*)	1682	5293	6975	760	2298	3058	922	2995	3058
	Total (*n*)	3482	6122	9604	1639	2788	4427	18.43	3334	4427
	PPA (%, 95% CI)	51.7 (50.0–53.4)			53.6 (51.2–56.0)			50.0 (47.7–52.3)		
	NPA (%, 95% CI)	86.5 (85.6–87.3)			82.4 (81.0–83.8)			89.8 (88.8–90.8)		
	OPA (%, 95% CI)	73.9 (73.0–74.7)			71.8 (70.4–73.1)			75.6 (74.5–76.8)		
ApoB ≥ 130 mg/dL	Dyslipidemia (*n*)	908	1721	2629	434	935	1369	474	786	1260
	Normal (*n*)	150	6825	6975	74	2984	3058	76	3841	3917
	Total (*n*)	8546	1058	9604	508	3919	4427	550	4627	5177
	PPA (%, 95% CI)	85.8 (83.6–87.8)			85.4 (82.1–88.2)			86.2 (83.0–88.8)		
	NPA (%, 95% CI)	79.9 (90.0–80.7)			89.5 (88.9–90.1)			83.0 (81.9–84.1)		
	OPA (%, 95% CI)	80.5 (79.7–81.3)			89.3 (88.7–89.9)			83.3 (82.3–84.3)		
ApoB ≥ 145 mg/dL	Dyslipidemia (*n*)	492	2137	2629	228	1141	1369	264	996	1260
	Normal (*n*)	19	6956	6975	9	3049	3058	10	3907	3917
	Total (*n*)	9093	511	9604	237	4190	4427	274	4903	5177
	PPA (%, 95% CI)	96.3 (94.3–97.6)			96.2 (92.9–98.0)			96.4 (93.4–98.0)		
	NPA (%, 95% CI)	76.5 (75.6–77.4)			72.8 (71.4–74.1)			79.7 (78.5–80.8)		
	OPA (%, 95% CI)	77.6 (76.7–78.4)			74.0 (72.7–75.3)			80.6 (79.5–81.6)		

Abbreviations: ApoB, apolipoprotein B; CI, confidence interval; NPA, negative percent agreement; OPA, overall percent agreement; PPA, positive percent agreement.

## Data Availability

The datasets generated and analyzed during the current study are available from the corresponding authors on reasonable request. The data are not publicly available due to privacy or ethical restrictions.
